# Deoxypodophyllotoxin Induces G2/M Cell Cycle Arrest and Apoptosis in SGC-7901 Cells and Inhibits Tumor Growth *in Vivo*

**DOI:** 10.3390/molecules20011661

**Published:** 2015-01-20

**Authors:** Yu-Rong Wang, Yuan Xu, Zhen-Zhou Jiang, Mounia Guerram, Bin Wang, Xiong Zhu, Lu-Yong Zhang

**Affiliations:** 1 Jiangsu Center for Drug Screening, China Pharmaceutical University, Nanjing 210009, China; E-Mails: yurong1987213@163.com (Y.-R.W.); xjwshxy@126.com (Y.X.); mouniaphar@yahoo.fr (M.G.); abinguolin2014@163.com (B.W.); 2 Jiangsu Center for Pharmacodynamics Research and Evaluation, China Pharmaceutical University, Nanjing 210009, China; 3 Medical and Chemical Institute, China Pharmaceutical University, Nanjing 210009, China; E-Mail: cpuzx@foxmail.com; 4 State Key Laboratory of Natural Medicines, China Pharmaceutical University, Nanjing 210009, China

**Keywords:** deoxypodophyllotoxin, human gastric cancer, cell cycle arrest, apoptosis, anti-angiogenesis

## Abstract

Deoxypodophyllotoxin (DPT), a natural microtubule destabilizer, was isolated from *Anthriscus sylvestris*, and a few studies have reported its anti-cancer effect. However, the* in vivo* antitumor efficacy of DPT is currently indeterminate. In this study, we investigated the anti-gastric cancer effects of DPT both* in vitro* and* in vivo*. Our data showed that DPT inhibited cancer cell proliferation and induced G2/M cell cycle arrest accompanied by an increase in apoptotic cell death in SGC-7901 cancer cells. In addition, DPT caused cyclin B1, Cdc2 and Cdc25C to accumulate, decreased the expression of Bcl-2 and activated caspase-3 and PARP, suggesting that caspase-mediated pathways were involved in DPT-induced apoptosis. Animal studies revealed that DPT significantly inhibited tumor growth and decreased microvessel density (MVD) in a xenograft model of gastric cancer. Taken together, our findings provide a framework for further exploration of DPT as a novel chemotherapeutic for human gastric cancer.

## 1. Introduction

Gastric cancer is a leading cause of death worldwide, accounting for approximately 700,000 deaths per year. Although surgery is the mainstay of any curative treatment, recurrences and metastases are still observed in approximately two-thirds of patients [1]. Since gastric cancer is not sensitive to radiotherapy, chemotherapy remains the most effective treatment for improving patients’ quality of life and prolonging their survival [2].

Microtubules, built by α/β-tubulin dimers, are key components of the cytoskeleton and associated with multiple cell functions [3]. During mitosis, microtubules undergo rapid polymerization and depolymerization to enable movement of chromosomes. As cell cycle progression approaches metaphase, microtubules are disrupted to form a mitotic spindle to facilitate chromosomal alignment on the metaphase plate. In this process, tubulin subunits freely exchange on the microtubules. If suchfree exchange of tubulin subunits is disrupted, the mitotic spindles will be compromised, thus interfering disturbing the cell division. Drugs, such as taxol and vinblastine, can bind tubulin and prevent its incorporation into growing microtubules. Consequently, cells undergoing division, especially those showing a rapid division, are killed. Anti-microtubule drugs constitute an important major class of anticancer drugs with broad activities both in solid tumors and hematological malignancies [4,5,6]. Due to the clinical drug resistance observed in patients using anti-microtubule drugs, the discovery of new agents with optimal biopharmaceutical and pharmacological properties becomes the focus of numerous academic and industrial groups [7].

**Figure 1:**
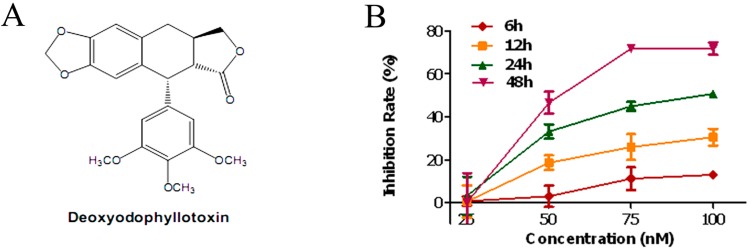
Antiproliferative activity of deoxypodophyllotoxin (DPT). (**A**) Chemical structure of DPT. (**B**) SGC-7901 cells were treated with different concentrations of DPT (25–100 nM) for the indicated time periods. Cell proliferation was then measured using a Cell Counting Kit-8. Data are presented as means ± SD of three independent experiments.

Previous studies showed that deoxypodophyllotoxin (DPT, Figure 1A), a naturally occurring flavolignan, inhibited microtubule assembly [8]. It shows potent antiproliferative and antitumor properties on several cancer types [9,10,11]. However, the* in vivo* antitumor efficacy of DPT are currently indeterminate and the details of the cellular and molecular mechanisms underlying its action against gastric cancer have not been systematically investigated. The present study aims to investigate the anti-gastric cancer effects of DPT both* in vitro* and* in vivo*, and to further characterize its mechanism.

## 2. Results

### 2.1. DPT Inhibited the Proliferation of SGC-7901 Cells

To determine the effect of DPT on cell proliferation, cells were treated with various concentrations of DPT for the indicated time periods and cell proliferation was determined using the CCK-8 assay. Proliferation of SGC-7901 cells was markedly inhibited by DPT-treatment in a dose- and time-dependent manner (Figure 1B). Taxol was used as positive control. The inhibition rates after taxol-treatment (10, 100 and 1000 nM) for 48 h were 18.24%, 49.35% and 78.02% respectively (data not shown).

### 2.2. DPT Destabilized Microtubules Assembly

**Figure 2:**
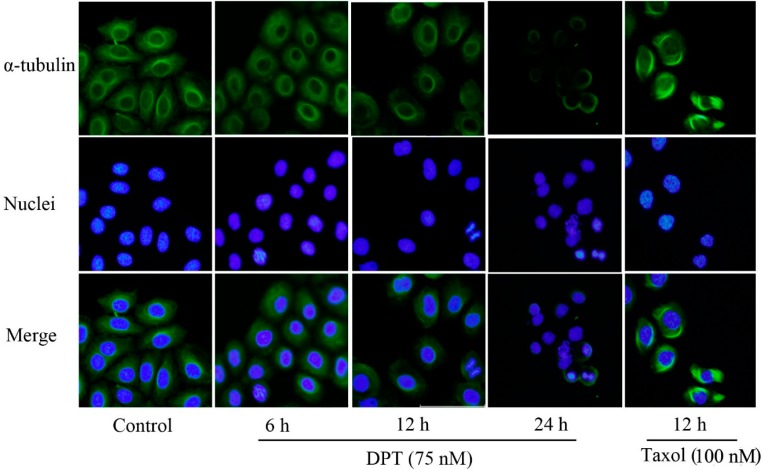
Disruption of microtubule (assembly) in DPT-treated SGC-7901 cells. Cells were treated with 75 nM DPT or 100 nM taxol for the indicated time courses. After fixation, cells were incubated with anti-α-tubulin antibody followed by Alexa-Fluor 488-conjugated secondary antibody and stained with Hoechst 33342 to visualize DNA (magnification: 200×).

The effect of DPT on microtubule dynamics was examined using anti-α-tubulin antibody. As shown in Figure 2, thin bundles of microtubules were distributed throughout the cytoplasm of the untreated cells.

In contrast, after DPT treatment for 6 h or 12 h, cells became round and contained short, dense microtubule networks. Cell shrinkage and nuclear fragmentation and condensation were observed after 24 h of DPT-treatment. Taxol resulted in a distinctive microtubule bundles that were likely due to the stabilization of rigid microtubule network.

### 2.3. DPT Induced Apoptosis in SGC-7901 Cells

**Figure 3:**
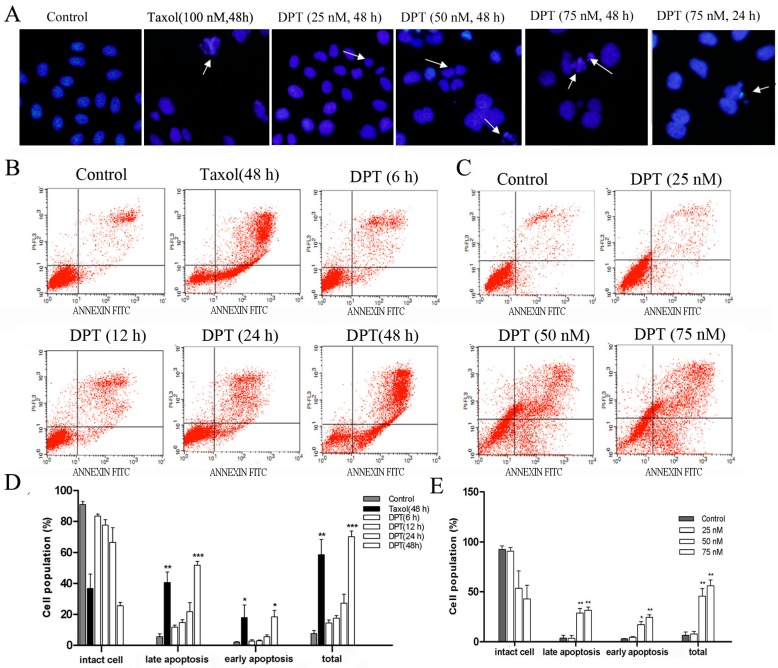
DPT induced apoptosis in gastric cancer cells. Cells were treated with different concentrations of DPT or taxol (100 nM) for the indicated time periods. (**A**) After DPT treatment, DNA was stained with Hoechst 33342 and observed under a fluorescence microscope (Magnification: 200×). Arrows indicate nuclear fragmentation in apoptotic cells. (**B**) Annexin V-FITC/PI double-staining assay showed the percentage of apoptotic cells after treatment with 75 nM of DPT or 100 nM of taxol for the indicated time and (**C**) after treatment with different concentrations of DPT for 48 h. (**D**,**E**) Statistical analysis of the number of apoptotic cells. Data are presented as means ± SD of three independent tests. * *p* < 0.05* versus* control, ** *p* < 0.01 *versus* control, *** *p* < 0.001* versus* control.

As shown in Figure 3B, DPT-treated cells showed a significant increase in the apoptotic cell population compared to the untreated cells. This effect was also time- and dose- dependent.

Indeed, treatment with DPT (75 nM) for 24 h and 48 h resulted in an increase in the percentage of early apoptotic cell population (lower right quadrant) from 2.05 to 5.62 and 18.49%, respectively. Meanwhile, treatment with doses of 25 nM and 50 nM increased the early apoptotic cell population from 3.17 to 4.00 and 20.20%. After treatment with 25, 50 or 75 nM of DPT for 48h and 75 nM of DPT for 24 h, the late apoptotic and necrotic cells (right upper quadrant) represented in 6.32%, 33.26%, 34.75% and 54.91% of total cells, whereas the control had only 4.31% necrotic cells.

Furthermore, DPT-treated SGC-7901 cells stained with Hoechst 33342 showed apoptotic changes, such as nuclear fragmentation and chromatin condensation (Figure 3A).These data were consistent with the induction of apoptosis by DPT.

### 2.4. DPT Induced G2/M Cell Cycle Arrest in SGC-7901 Cells

**Figure 4:**
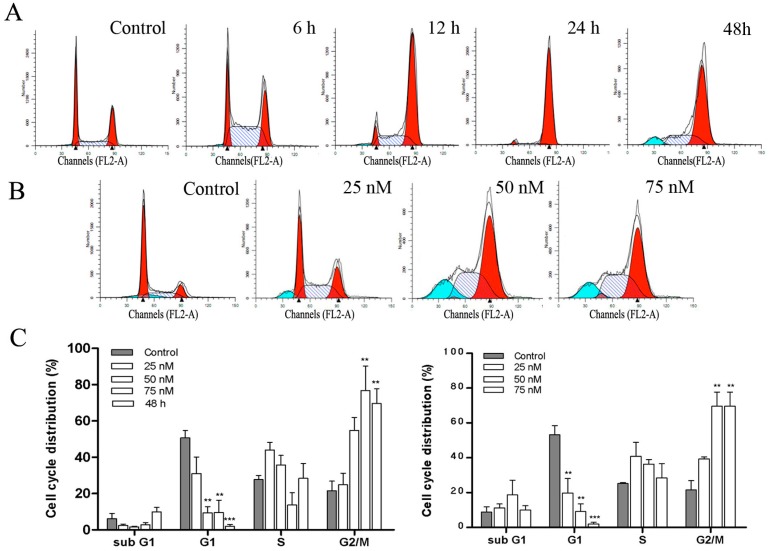
Effect of DPT on cell cycle distribution in SGC-7901 cells. Cell cycle distribution was monitored by flow cytometry. (**A**) PI staining assay was performed after DPT-treatment (75 nM) for the indicated time periods. (**B**) PI staining assay was performed after treatment with different concentrations of DPT for 48 h. (**C**) Statistical analysis of cell cycle phase distribution. Data are presented as means ± SD of three independent tests. ** *p* < 0.01* versus* control, *** *p* < 0.001* versus* control.

In order to determine the effect of DPT on cell cycle progression, flow cytometry analysis was performed on cells treated with or without DPT for various lengths of times. The results showed that DPT-treated SGC-7901 cells were arrested in G2/M phase in time- and dose- dependent manners (Figure 4). This was accompanied by a significant decrease in the G1 phase compared to the untreated control cells.

### 2.5. Effects of DPT on the Expression of Cell Cycle Regulatory Proteins

**Figure 5:**
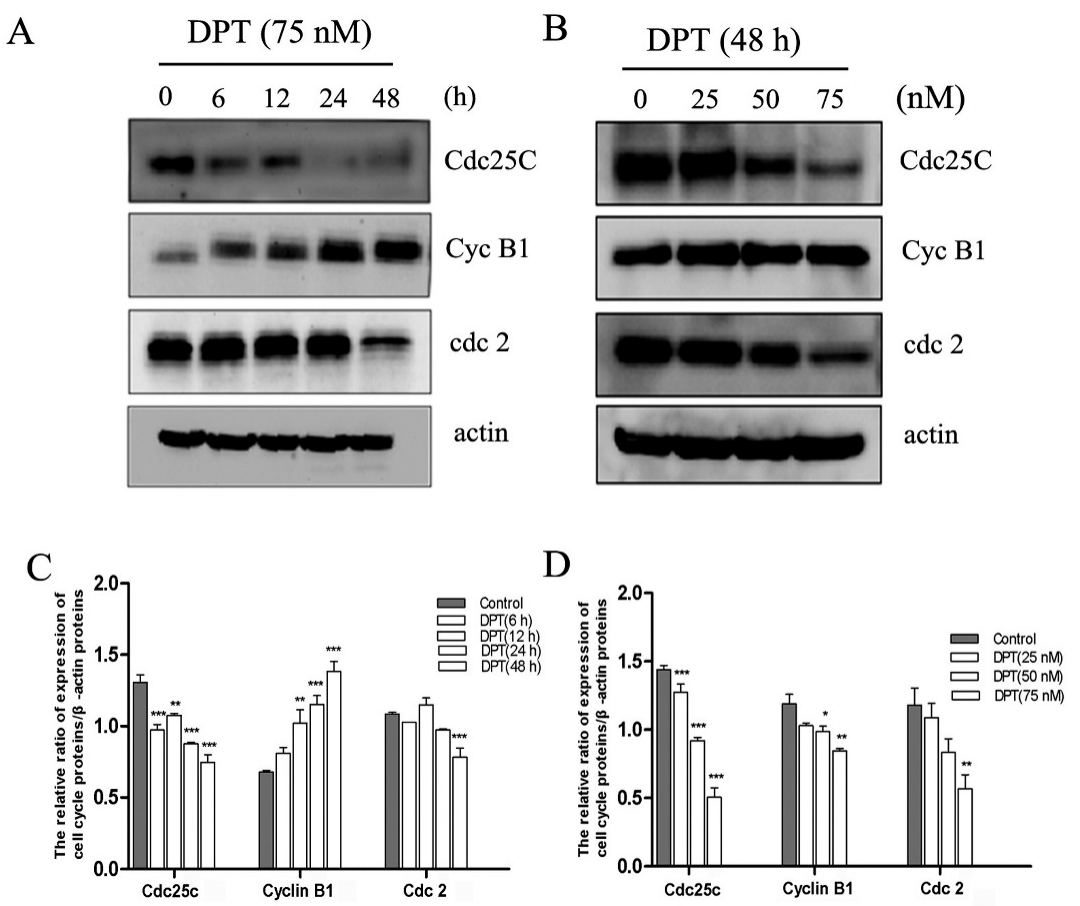
Effects of DPT on cell cycle regulatory proteins in SGC-7901 cells. The levels of cell cycle-related proteins including Cdc25C, cyclin B1 and Cdc2 were assessed by western blot analysis. (**A**) Cells were treated with DPT (75 nM) for the indicated time courses. (**B**) Cells were treated with different concentrations of DPT for 48 h. (**C**,**D**) Statistical analysis of cell cycle arrest relating-proteins. Data are presented as means ± SD of three independent tests. * *p* < 0.05* versus* control, ** *p* < 0.01* versus* control, *** *p* < 0.001* versus* control.

To further characterize the mechanism by which DPT induced G2/M cell cycle arrest, we examined the effects of DPT on the expression of cyclin B1, Cdc2 and Cdc25C proteins. As shown in Figure 5, the cellular level of cyclin B1 significantly increased within 6h after DPT-treatment and continued to increase up to 48 h. Furthermore, DPT-therapy resulted in a remarkably time- and dose-dependent decrease in Cdc2 and Cdc25C expression levels. These data indicate that DPT may induce G2/M arrest by altering the expression of cyclin B1, Cdc2 and Cdc25C proteins.

### 2.6. DPT Induced Caspase Activation and Inactivation of Bcl-2 Protein

**Figure 6:**
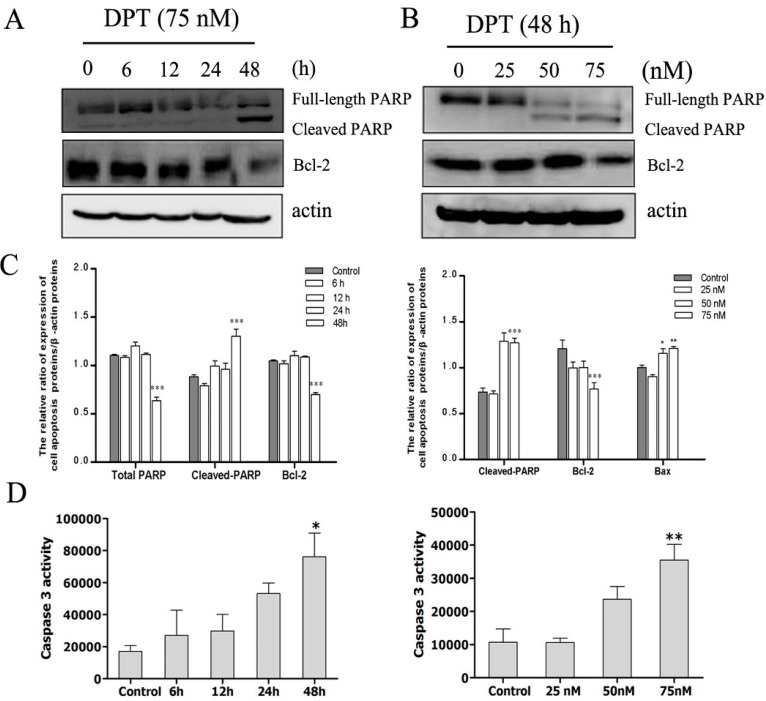
Effects of DPT on apoptotic-related protein and caspase-3 activity in SGC-7901 cells. The levels of PARP and Bcl-2 were assessed by western blot analysis. Capase-3 activity was evaluated by the detection of the cleavage of a colorimetric caspase-3 substrate, N-acetyl-Asp-Glu-Val-Asp (DEVD)-p-nitroaniline. (**A**) Cells were treated with 75 nM of DPT for the indicated time periods. (**B**) Cells were treated with different concentrations of DPT for 48 h. (**C**) Statistical analysis of PARP and Bcl-2. (**D**) Statistical analysis of caspase-3 activity. Data are presented as means ± SD of three independent tests. * *p* < 0.05* versus* control, ** *p* < 0.01* versus* control, *** *p* < 0.001* versus* control.

A Caspase-3 Activity Assay Kit was used to determine whether caspase family is involved in DPT-induced apoptosis. As presented in Figure 6D, the activity of caspase-3 was significantly increased after treatment with 75 nM of DPT for 24 h and 48 h. Treatment with 25 nM or 50 nM of DPT for 48 h also resulted in a remarkably increase in the activity of caspase-3.

It is well-known that caspase-3 and caspase-7 catalyze the processing of native 113-kDa PARP to the 89-kDa and 24-kDa. The amount of this typical cleaved form of PARP was increased after DPT-treatment in a time- and dose- dependent manner (Figure 6A–C). Collectively, these results indicated that the induction of apoptotic cell death by DPT probably occurred through a caspase-dependent pathway after G2/M cell-cycle arrest, and was observed after approximately 24 h of DPT exposure.

To further elucidate the mechanism involved in DPT-mediated apoptosis, we measured the expression of Bcl-2 protein which was associated with the outer mitochondrial membrane. Treatment with DPT resulted in a dose- and time- dependent decrease in the expression of the anti-apoptotic Bcl-2 protein (Figure 6C).

### 2.7. DPT Possessed Potent Anticancer Activity in Vivo

**Figure 7:**
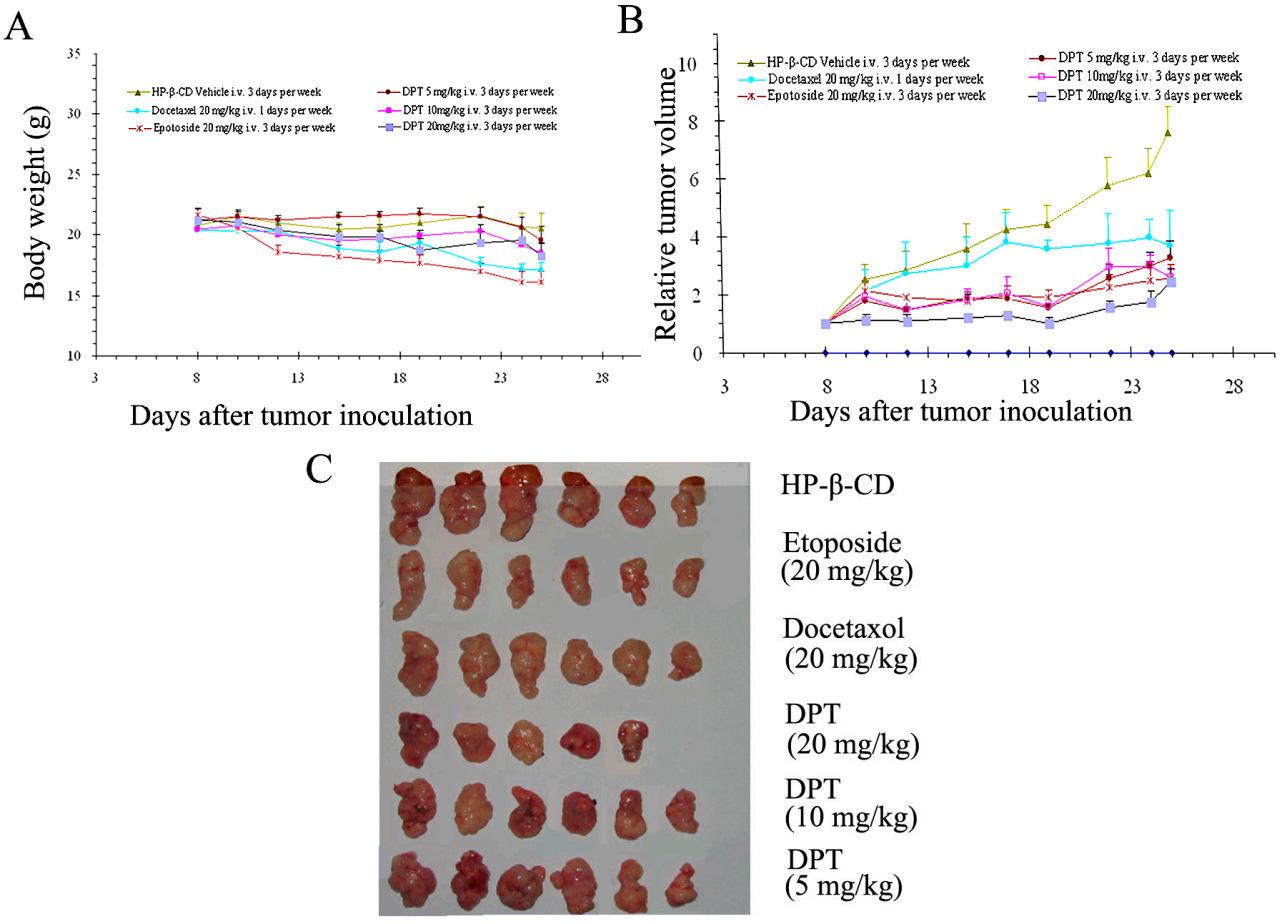
Effects of DPT on the growth of SGC-7901 tumor in nude mice (n = 6). Tumor diameter was serially measured with a vernier caliper, and the relative tumor volume was calculated by the equation described in Section 2. The body weight changes of animals with SGC-7901(xenografts) (**A**) and relative tumor volume (**B**) are shown.

Since DPT is water-insoluble, an HP-β-CD inclusion complex (containing 3.06% of DPT) was prepared to further characterize its antitumor activity* in vivo*. Mice were treated with vehicle (HP-β-CD inclusion complex), 5, 10, and 20 mg/kg of DPT three times a week. Comparisons were made with a single dose of docetaxel (20 mg/kg) injected one time a week and 20 mg/kg etoposide injected three times a week. DPT significantly suppressed the tumors derived from gastric cancer SGC-7901 cells in a dose-dependent manner (Figure 7A). Table 1 revealed that the growth of tumors was inhibited by 22.19%, 47.91% and 50.93% with DPT at 5, 10 and 20 mg/kg, respectively, compared with that of vehicle-treated animals, respectively. At high dose, the inhibitory effect of DPT was similar to that of positive drug etopside, which produced the inhibition ratio of 53.11 and was higher than docetaxol, which produced the inhibition ratio of 42.63. No significant weight loss was observed in treated groups (Figure 7B); however, one animal receiving DPT-therapy at high dose of 20 mg/kg died.

**Table 1:** Inhibitory effects of DPT on the tumor growth of SGC-7901 in nude mice.

Group	Dosage (mg/kg)	Tumor Weight (g)	Inhibition Rate (%)
Docetaxel	20	0.83 ± 0.12 *	42.63
Etoposide	20	0.68 ± 0.16 **	53.11
DPT	5	1.12 ± 0.03 *	22.19
	10	0.75 ± 0.06 *	47.91
	20	0.71 ± 0.11 *	50.93

Data are expressed as means ± SEM (n = 6); * *p* < 0.05* versus* control, ** *p* < 0.01* versus* control. Significant difference was calculated by one-way ANOVA.

### 2.8. DPT Decreased Microvessel Density (MVD)

**Figure 8:**
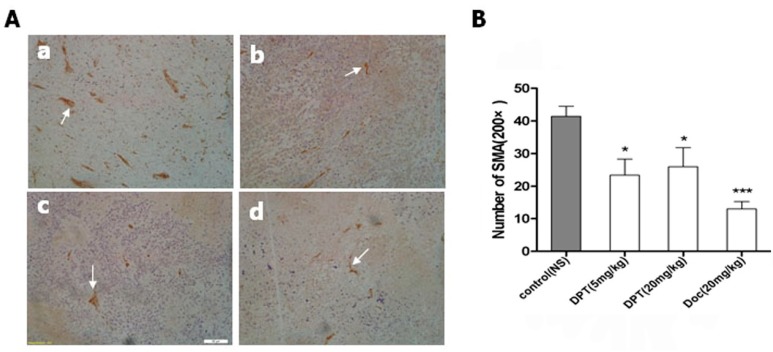
Antiangiogenic effects of DPT on SGC-7901 gastric cancer xenograft. Animals were treated with control, DPT or docetaxel. (**A**) Immunohistochemical analysis of blood vessels (arrow) in tumor tissue from treated animals: (**a**) control; (**b**) Docetaxel, 20 mg/kg; (**c**) DPT, 5 mg/kg; (**d**) DPT, 20 mg/kg. (**B**) Dose-response effect of DPT and docetaxel on tumor vascular density. Each point represents mean ± SEM (n = 3) from a representative experiment.

The mean MVD counts of tumors derived from SGC-7901 gastric cells were 41.40 ± 7.02 in the group treated with HP-β-CD inclusion complex, 13.00 ± 4.95 in the group treated with docetaxel, 23.40 ± 10.99 and 23.60 ± 13.02 in the groups treated with 5 mg/kg DPT and 20 mg/kg DPT, respectively. Figure 8A(b–d) showed a representative field of low MVD in tumors derived from SGC-7901. For comparison, Figure 8A(a) depicted a representative field with high MVD.

## 3. Discussion

Natural products, especially microtubule-binding agents, play important roles in the fight against cancer. From the clinical use of vinblastine in 1961 and paclitaxel in 1992 to ixabepilone in 2007, microtubule-binding natural products have continually contributed to the development of cancer therapy. Clinically, inhibiting microtubules is the primary therapeutic strategy for the treatment of gastric cancer. Furthermore, the clinical success of the currently available microtubule-binding chemotherapeutic agentsis mainly based on their direct and strong cytotoxic effects against tumor cells. DPT, a derivative of podophyllotoxin, is a lignan with potent antimitotic and antiviral properties isolated from rhizomes of *Sinopodophullum hexandrum* (Berberidaceae). Several studies indicated that DPT inhibits microtubule assembly and cell growth of several types of human cancer cell lines. DPT has been found to regulate gene expression associated with cell proliferation, cancer cell invasion and metastasis* in vitro* [12,13,14]. In the present study, we established a xenograft model of gastric cancer in nude mice and systematically evaluated the anti-gastric cancer effects of DPT both* in vitro* and* in vivo*.

Our* in vitro* investigations confirmed that DPT treatment induced extensive microtubule depolymerization and disrupted the microtubule network in SGC-7901 cancer cells, compared to the effects observed in cells treated with taxol. Results from CCK-8 assay showed that DPT significantly suppressed SGC-7901 cell proliferation and viability in a dose- and time- dependent manner.

In accordance with the fact that microtubule-binding agents arrest the cell cycle at G2/M phase [15], our results showed that DPT induced a G2/M blockade in SGC-7901 cells as indicated by flow cytometry analysis. We further investigated the molecular mechanism by which DPT stopped the cell cycle. It is well-known that cyclins and cyclin-dependent protein kinases (Cdks) are key regulators of the cell cycle progression. Cyclin B1 plays animportant role in the G2/M transitionas well as in the M phase progression. As reported, the cyclin B1 protein level substantially accumulates in G2 phase, peaks as cell division approaches metaphase, and rapidly decreased during anaphase [16,17]. We observed an accumulation of cyclin B1 protein 6 h after DPT-treatment. Its cellular level considerable increased after 48 h of treatment. It is well-known that cyclin B1 accumulation is a marker of cells stopped in G2 and/or M phases of the cell cycle. Cdc2 interacts with cyclin B1 and form the maturation promoting factor (MPF) which regulates the transition from G2 to M phase [18]. Cdc25C regulates the subsequent activation of cyclin B1/Cdc2 complex by removing the inhibitory phosphorylations of Cdc2 on Thr14/Tyr15. In the present study, the expression of Cdc2, Cdc25C regulatory proteins markedly changed after administration of DPT, implying that those proteins may be involved in DPT-induced G2/M arrest.

Cells would either undergo repair mechanisms or follow the apoptotic pathway when the arrest of cell cycle progression at G2 phase occurs. FACS analysis showed a marked accumulation of SGC-7901 cells in G2/M phase prior to the induction of a sub-G1 cell population. These data suggest that G2/M-phase arrest might be an upstream event leading to apoptosis. The fact that blockage of cell cycle progression at mitotic phase leads to apoptotic cell death has been well established [6]. In the present study, the Annexin V/PI double-staining assay showed that the apoptotic rate of SGC-7901 cells significantly increased after DPT treatment.

The apoptosis signal is mainly regulated by the caspase family, which exist as inactive zymogens in cells and undergo a cascade of catalytic effects at the initiation of apoptosis. Activation of caspase-3 and caspase-7 leads to the cleavage and inactivation of many cellular proteins, such as lamin and PARP, therefore resulting in apoptotic cell death in many cell types [19]. Our results showed that DPT induced activation of caspase-3 which was accompanied by PARP cleavage, indicating that the caspase apoptotic pathway is involved in the mechanism of DPT-induced cell death.

The strong tumor inhibition properties as well as the caspase-mediated apoptotic action of DPT prompted us to evaluate its efficacy and safety* in vivo*. We used the well-known anti-cancer agents docetaxel and etopside as positive controls. Docetaxel was chosen due to the fact that it is widely used in the clinic as a first-line drug for gastric cancer chemotherapy and shares the similar mechanism of DPT action. On the other hand and like DPT, etopside is a derivative of phodophyllotoxin. In our preliminary experiments, DPT suppressed tumor growth at a dose of 20 mg/kg to an extent without any significant changes of the mice body weight (Figure 7A,B). Side effects, such as hair loss, lethargy, dysphoria, or other macroscopicalvisceral pathogenic changes were not observed (data not shown). DPT at 5 and 10 mg/kg also exerted robust growth inhibitory activity in the xenograft model. In contrast, marked weight loss was observed in the mice administrated etopside. Furthermore, the effect of DPT at 10 and 20 mg/kg was more pronounced than that of docetaxel. Hence, these data clearly indicated that DPT possessed a strong anti-tumor activity* in vivo* with a reasonable safety margin.

The targets of microtubule-binding agents in cancer therapy include both cancer cells and vascular endothelial cells. Angiogenesisis mediated by endothelial cells can be quantified through counting microvasculars in a given area by immunohistochemical staining (microvessel density, MVD) [20]. Indeed, MVD has been extensively evaluated as a measure of angiogenesis [21]. MVD in tumors derived from SGC-7901 cells xenograft significantly decreased after DPT and docetaxel treatment for three week. Consequently, our results provide the initial evidence that DPT exerts a potent anti-angiogenic effect.

Antiangiogenesis has been an attractive anticancer strategy for more than fifty years [22]. Compounds that target the microtubule have been greatly successful in the clinic as chemotherapeutics, and this success is likely due to their ability to target cells regardless of their cell cycle stage. Preclinical and clinical studies have suggested that microtubule-binding agents might be a particularly useful class of drugs for vascular-targeted therapy [23,24].There are a lot of these compounds in development that act on the vasculature, and various formulations of clinically used drugs are being developed to take advantage of these anti-angiogenic properties. Thus, DPT, as a drug that target the microtubule will continue to have a major impact in oncology not only as anti-mitotics but also as potent inhibitors of angiogenesis.

## 4. Experimental

### 4.1. Materials

DPT and it’s hydroxypropyl-β-cyclodextrin (HP-β-CD) inclusion complex were obtained from the Medicinal Chemical Institute, China Pharmaceutical University, Nanjing, China. Taxol was purchased from Guilin Huiang Biochemistry Pharmaceutical Ltd (Guilin, Guangxi, China). Docetaxol and etopside were obtained from Qilu Pharmaceutical Ltd (Jinan, Shandong, China). A stock solution (10^−2^ M) was prepared in DMSO and stored at −20 °C. The antibodies against β-actin and α-tubulin were purchased from Santa Cruz Biotechnology (Santa Cruz, CA, USA). The antibodies against cyclin B1, Cdc2, Cdc25C, PARP and Bcl-2 were purchased from Cell Signaling Technology (Beverly, MA, USA). Alexa-Fluor 488 (green)-conjugated second antibody was purchased from Invitrogen (Carlsbad, CA, USA). Cell cycle and Apoptosis Analysis Kit(s), Caspase-3 Activity Assay Kit and Hoechst 33342 were purchased from Beyotime Institute of Biotechnology (Suzhou, China). FITC-Annexin V Apoptosis Detection Kit was purchased from BD Bioscience (San Diego, CA, USA). Cell Counting Kit (CCK-8) was purchased from Dojindo Laboratories (Kumamoto, Japan).

### 4.2. Cell Culture

Human gastric carcinoma SGC-7901 cell line was purchased from Type Culture Collection of Chinese Academy of Sciences, Shanghai, China. Cells were cultured in RPMI-1640 medium supplemented with 10% fetal bovine serum, 100 U/mL penicillin and 100 μg/mL streptomycin (all reagentswere purchased from Hyclone (Logan, UT, USA) and maintained in a humidified atmosphere containing 5% CO_2_.

### 4.3. Animals

Female nude mice (6–8 week old, BALB/c) were used to establish the SGC-7901 xenograft tumor model and were purchased from the Institute of Laboratory Animal Science, Academy of Military Medical Sciences of the Chinese PLA (Beijing, China). All animal tests and experimental procedures were approved by the Ethical Committee of China Pharmaceutical University, Nanjing University, and Laboratory Animal Management Committee of Jiangsu Province (Approval ID: 2110682).

### 4.4. Immunofluorescence Analysis

SGC-7901 cells were plated on glass coverslips and treated with vehicle, 75 nM DPT, or with 100 nM taxol [12] diluted in media for 6, 12 and 24 h. The cells were fixed with 4% paraformaldehyde for 30 min, permeabilized in 0.1% Triton X-100/TBS for 10 min, blocked with 5% bovine serum albumin for 1 h to reduce nonspecific staining and then incubated with primary anti-α-tubulin antibody (4 °C, overnight) and Alexa-Fluor 488-conjugated secondary antibody for 60 min. The nucleus was stained with Hoechst 33342 for 1 h. Fluorescence images were obtained using a confocal microscope (FV-1000; Olympus, Tokyo, Japan).

### 4.5. Cell Proliferation Analysis

Cells were seeded in 96-well plates at a density of 1500–3000 cells/well and allowed to adhere overnight and then treated with either vehicle (RPMI-1640 medium) or DPT (25, 50, 75 or 100 nM) for 6, 12, 24, 48 and 72 h. Cell proliferation was assessed using the CCK-8 assay [25]. The inhibition rate of cell proliferation was calculated as (OD_control_ − OD_treated_)/OD_control_ × 100%.

### 4.6. Cell Cycle Analysis

SGC-7901 cells were treated with DPT (25, 50 and 75 nM) for 6, 12, 24 and 48 h in complete medium. The floating and adherent cells were collected, washed twice with cold PBS and centrifuged. Cells were then fixed in 70% (*v*/*v*) ethanol for 24 h at 4 °C. After centrifugation, cells were washed with cold PBS and stained according to the manufacturer’s protocol. After incubation for 30 min in the dark, cell cycle distribution was determined by flow cytometry (BD, FACSCalibur) using multi-cycle Software (ModFit LT 3.2 Mac).

### 4.7. Apoptosis Detection

SGC-7901 cells were treated with DPT as previously described for various lengths of time, harvested, washed with cold PBS, and stained with AnnexinV-FITC/PI according to the manufacturer’s protocol. After incubation, the apoptotic cells were measured by flow cytometry (BD, FACSCalibur) using the CELLQUEST Pro.

### 4.8. Western Blot Analysis

Cells were treated with DPT and cell lysates were prepared as described previously [26]. Equal amounts of cell lysates (50 µg of protein) were resolved by SDS-polyacrylamide gel electrophoresis and electrophoretically transferred onto polyvinylidenedifluoride membranes (PVDF) (Millipore; Bedford, MA, USA). Membranes were then blocked with 5% non-fat dry milk in Tris-Buffed- Saline with Tween (TBST) for 1 h, and incubated with appropriate dilutions of primary antibodies (overnight, 4 °C) followed by horseradish peroxidase-conjugated secondary antibodies. The immunoreactive proteins were then detected by the ECL-Plus Western Blotting Detection System.

### 4.9. Caspase-3 Activity Assay

Caspase-3 activity was assayed by colorimetric detection of p-nitroanilidine (pNA) after cleavage of the peptide substrate, N-acetyl-Asp-Glu-Val-Asp (DEVD)-*p*-nitroaniline, specific for caspase-3. Gastric cancer cells were treated with DPT as described previously. Floating and adherent treated-cells were then collected and lysed in ice-cold lysis buffer for 15 min. Absorbance was measured at 405 nm with a Tecan microplate reader (Safire2, TECAN, Männedorf, Switzerland) after 12 h of incubation in a humidified atmosphere of 5% CO_2_ in air at 37 °C. Data are presented as mean ± SD of three independent experiments.

### 4.10. In Vivo Study

Xenograft model of gastric cancer was established by a subcutaneous injection (*s.c.*) of 3 × 10^6^ SGC-7901 cells into the right rear flank of each mouse. Following two weeks of growth, tumor tissues were cut into multiple 3 × 3 × 3 mm^3^ pieces and implanted (*s.c.*) into the right rear flank of each mouse using a range trocar. Tumor diameters were measured with a caliper and tumor volume was calculated by the formula: Volume = (width)^2^ × length/2. Treatments were started after one week when the tumors reached an average volume of 100–200 mm^3^. Animals were randomly divided into 6 groups (n = 6) and intravenously injected with: (a) HP-β-CD; (b) 20 mg/kg docetaxel; (c) 20 mg/kg etoposide; (d) 5 mg/kg DPT; (e) 10 mg/kg DPT; (f) 20 mg/kg DPT. HP-β-CD, DPT and etoposide were administrated three times a week and docetaxel was administrated once a week. After 21 days of treatment, mice were sacrificed and tumors were weighed and excised for immunohistochemistry assay. The inhibition rate was calculated as: (tumor weight of vehicle control group − tumor weight of treatment group)/tumor weight of vehicle control group × 100%.

### 4.11. Quantification of Microvessel Density

Microvessel density (MVD) in tumors derived from SGC-7901 cell line xenograft was examined using a Blood Vessel Staining Kit (Millipore). Primary antibody against von Willebrand Factor was used to evaluate MVD and was performed according to the methods described previously [16]. MVD was assessed on vWF stained slides. Endothelial cell clusters and endothelial cells which were stained brownish-yellow were regarded as a single microvessel [27]. Microvessels were counted on five microscopic fields per specimen at ×200 magnification [28].

### 4.12. Statistical Analysis

All data represent mean values of at least three independent experiments and are expressed as mean ± SD. Statistically significant differences were assessed via one-way ANOVA followed by Tukey’s *post hoc* test for multiple comparison tests. A value of *p* < 0.05 was considered statistically significant.

## 5. Conclusions

In summary, this is the first study of DPT with potential anti gastric cancer both* in vitro* and* in vivo*. Our investigation reveals that DPT can destabilize microtubules, induces G2/M arrest by regulating proteins of cell cycle, such as cyclin B1, Cdc2 and Cdc25C and leads to apoptotic cell death through activation of caspase-3 and cleavage of PARP. We also demonstrated that DPT strongly and notably decreased the microvessel density in tumors derived from SGC-7901 cells xenografts. Therefore, our study provides a scientific support that DPT has a potential therapeutic value in the treatment of human gastric cancer.
